# No evidence for aversive associative olfactory memory through metamorphosis in *Drosophila*

**DOI:** 10.1242/bio.062512

**Published:** 2026-08-07

**Authors:** Haiko Poppinga, Rotem Mika, Maria Schwartz, Oren Schuldiner, Hagar Meltzer, André Fiala

**Affiliations:** ^1^ University of Göttingen, Department of Molecular Neurobiology of Behavior, Julia-Lermontowa-Weg 3, 37077 Göttingen, Germany; ^2^ Weizmann Institute of Science, Department of Molecular Cell Biology and Department of Molecular Neuroscience, Belfer Building of Life Sciences, Rehovot 7610001, Israel

**Keywords:** Neuronal remodeling, Larval memory, Mushroom body, Olfactory conditioning, Metamorphosis, Axon pruning, Associative aversive memory, Long-term memory

## Abstract

Do memories acquired through learning by insects in the larval stage persist throughout metamorphosis into adulthood? There are contradictory reports to answer this question. After a larval stage, holometabolous insects undergo pupation and metamorphosis, during which the body is substantially transformed. This remodeling also affects the brain: neuronal connections degrade, axons and dendrites prune and regrow, new neurons are generated, and new synaptic connections are established. Such drastic remodeling appears incompatible with reports from several insect species, including *Drosophila melanogaster*, suggesting that larval memories are maintained throughout metamorphosis and can be observed in the adult imago. We use aversive associative olfactory conditioning of wild-type and pruning-defective *D. melanogaster* larvae to revisit this long-standing and unresolved question. In contrast to previous reports, we found no evidence that memories acquired through classical aversive conditioning in the larval stage survive the neuronal remodeling processes during metamorphosis. Even when pruning and regrowth of larval Kenyon cells of the mushroom body, a central brain structure essential for associative learning, are largely prevented by genetic intervention, no maintenance of larval memories could be detected in the adult fly. We conclude that at least larval olfactory memories acquired through aversive classical conditioning are erased over the course of metamorphosis.

## Teaser

**Summary:** We investigated whether memories formed in *Drosophila melanogaster* larvae persist through metamorphosis and found that aversive odor learning in larvae is not retained in adult flies.

## INTRODUCTION

The primary function of the nervous system across animal taxa is to regulate behavior and enable adaptation to dynamic external and internal conditions. Central to this adaptive capacity are the processes of learning and memory, which rely on the plasticity of synaptic connections within neuronal circuits (Martin et al., 2000). A fundamental question in neuroscience concerns whether and how memories are maintained despite the continuous remodeling of neural tissue, including synaptic structures (Blackiston et al., 2015). This issue is particularly compelling in holometabolous insects, which undergo complete metamorphosis. Species such as flies and beetles experience profound morphological and behavioral transformations as they transition from larval to adult stages. While larvae primarily engage in foraging and feeding, adults often shift their behavioral priorities toward reproduction. Despite these life-stage differences, both larvae and adults are capable of learning and modifying behavior based on prior experience. This raises a central question: to what extent can memories acquired during the larval stage persist through metamorphosis into adulthood?

Research addressing this question has examined a wide range of insect species. For example, house flies (*Musca domestica*) reared in scented sawdust as larvae later exhibit a preference for those same scents as adults (Ray, 1999). Similarly, evidence for memory transfer across metamorphosis has been reported in beetles such as *Tenebrio molitor* and *Tenebrio obscurus* (Alloway, 1972; Alloway and Routtenberg, 1967; Borsellino et al., 1970; Punzo and Malatesta, 1988), in moths including *Manduca sexta* and *Grapholita molesta*, and in the parasitic wasp *Hyssopus pallidus* (Blackiston et al., 2008; Sant'Ana et al., 2021; Gandolfi et al., 2003).

Associative olfactory learning, often studied through Pavlovian conditioning (Pavlov, 1927), has been investigated in detail in *Drosophila melanogaster* flies at both larval and adult stages. When an odor is paired with a rewarding unconditioned stimulus such as sugar, flies and larvae reliably develop a preference for that odor (Tempel et al., 1983; Pauls et al., 2010a). Conversely, pairing an odor with an aversive stimulus, such as electric shock or a concentrated salt solution, induces odor avoidance (Aceves-Piña and Quinn, 1979; Niewalda et al., 2008; Khurana et al., 2009; Pauls et al., 2010b; Tully and Quinn, 1985; Widmann et al., 2018). Importantly, Tully et al. (1994) reported that an aversive olfactory memory acquired during the third-instar larval stage can be maintained across metamorphosis and expressed in the adult fly.

The neural circuits underlying these forms of associative learning are well characterized. In adult flies, odors are detected by olfactory sensory neurons in the antennae and maxillary palps (Galizia and Sachse, 2010), whereas in larvae they are detected by sensory neurons in the dorsal organ (Stocker, 2008). The olfactory receptors expressed in larvae and adults differ substantially (Kreher et al., 2005, 2008), but some overlap exists. Olfactory sensory neurons terminate in the antennal lobes – in larvae in the embryonic antennal lobes, which degenerate during pupariation and become replaced by the adult antennal lobes. In both stages, olfactory projection neurons convey information to higher brain centers, including the mushroom body, a critical locus for associative learning (Gerber and Stocker, 2007). The adult mushroom body is comprised of three types of Kenyon cells (KCs) – γ, α′/β′ and α/β – which together form five axonal lobes. Subsets of KCs are selectively activated by odors (Bilz et al., 2020). The coincidence of odor input (conditioned stimulus) with reinforcement (unconditioned stimulus) occurs in the axonal regions of KCs. These regions are defined by localized and segregated innervations by modulatory dopaminergic neurons (DANs) and mushroom body output neurons (MBONs) that together form, in the case of the adult γ axonal lobe, five distinct zones or compartments termed γ1-5. The larval γ lobes are similarly innervated in a compartmentalized fashion – three compartments of the vertical lobe and four of the medial lobe. Indeed, DANs provide the reinforcing signal for punishment during aversive learning, primarily via the PPL-1 cluster targeting the γ1 compartment in adults (Aso et al., 2014), while in larvae, equivalent processes are mediated by specific DANs (Schroll et al., 2006; Eschbach et al., 2020). Coincident activity between dopaminergic input and KCs induces synaptic plasticity at KC-to-MBON synapses, driving approach or avoidance behaviors (Aso et al., 2014; Eschbach et al., 2020, 2021). However, during metamorphosis, all components of this circuit undergo extensive restructuring – the γ-KCs, which are the main KC type at this developmental stage, undergo axon and dendrite pruning followed by regrowth to the adult medial lobe (Yaniv and Schuldiner, 2016), while MBONs and DANs undergo remodeling, which in some cases involves trans-differentiation (in which they project their processes to non-mushroom body regions) or even cell death (Truman et al., 2023). Remarkably, a comprehensive analysis of cell fate during mushroom body remodeling has concluded that no larval input-output pair is maintained (Truman et al., 2023). This profound reorganization raises the critical question of whether and how memories formed during the larval stage can persist into the adult brain.

## RESULTS

Associative olfactory learning in *D. melanogaster* larvae is well established, with both rewarding and aversive stimuli shown to induce robust behavioral modifications (Pauls et al., 2010a,b). Moreover, different memory phases, defined by distinct molecular cascades in adult flies, have also been identified in larvae (Widmann et al., 2016; Eschment et al., 2020). In particular, protein synthesis-dependent long-term memory (LTM) can be induced through multiple temporally spaced training trials, similar to adults, and is contingent on sufficient metabolic energy, which can be ensured by providing sucrose prior to training (Eschment et al., 2020). Based on these findings, we hypothesized that if an associative olfactory memory acquired during the third-instar larval stage were to persist across metamorphosis, it would most likely represent an LTM trace.

**Fig. 1.**
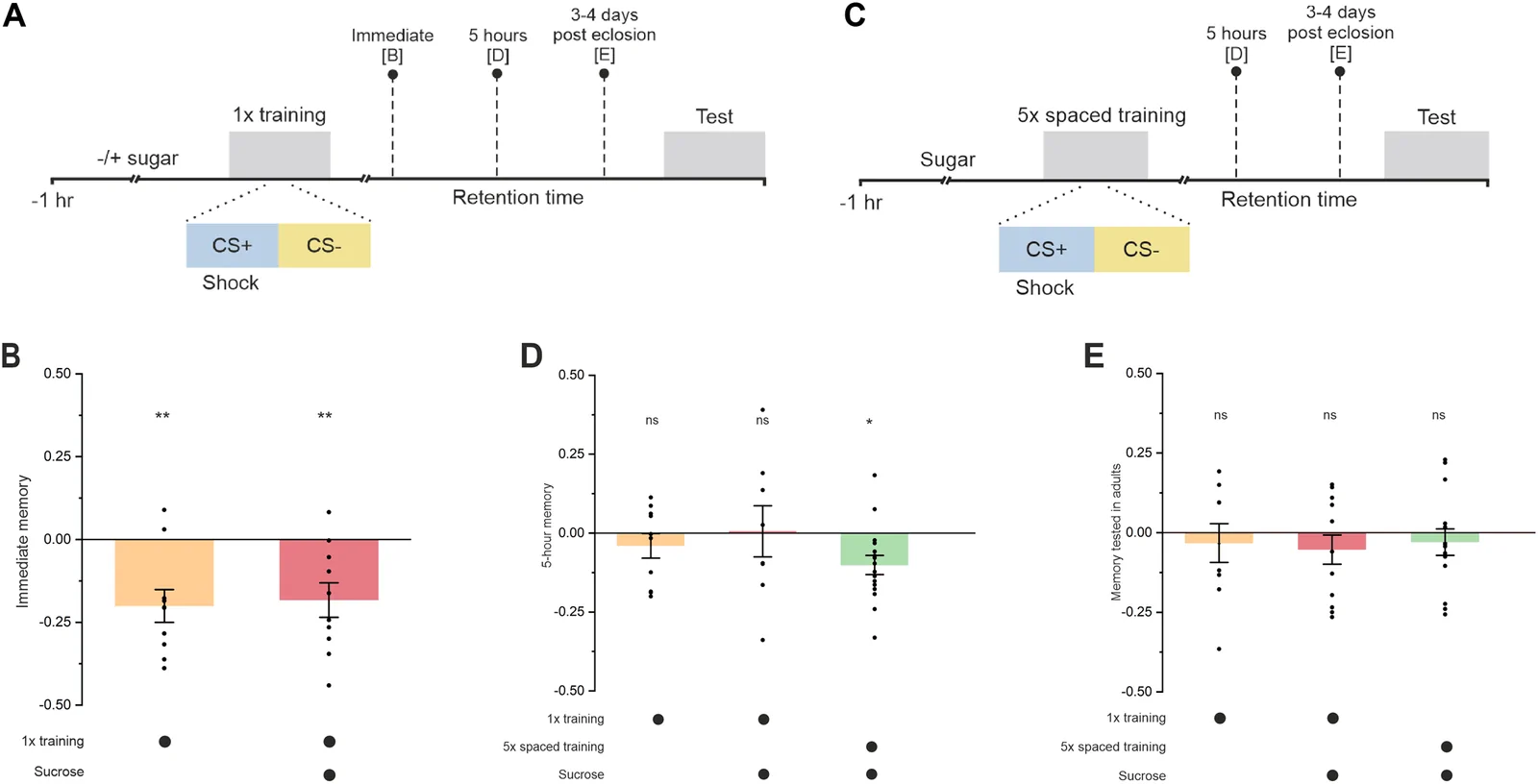
**No memory through metamorphosis in wild type *D. melanogaster* after olfactory electric shock conditioning**. (A) Schematic of single-cycle electric shock training (1× training) with or without 1-h sucrose feeding prior to training. During the training, one odor was paired with electric shock (CS+) and the other was not (CS−). Memory was tested at different time points after training (retention time) as written in the scheme (with the relevant panels indicated). (B) *D. melanogaster* larvae showed immediate memory (memory tested directly after training) after 1× electric shock training, both without (*n*=10) and with (*n*=10) sugar feeding (one-sample *t*-test with Holm-Sidak adjusted *P*-values; *P*=5.75×10^−3^, *P*=6.64×10^−3^, respectively). (C) Schematic of five-cycle electric shock spaced training (5× spaced training) with 1-h sucrose feeding prior to training. The training consists of five cycles, each separated by a 15-min rest period. Memory was tested at different time points after training (retention time) as written in the scheme (with the relevant panels indicated). (D) 5-h memory (memory tested after 5 h) is not detectable after 1× electric shock training without (*n*=10) or with (*n*=12) sugar feeding, but is detectable after 5× spaced training (*n*=16; one-sample *t*-test with Holm-Sidak adjusted *P*-values; *P*=0.546, *P*=0.758, *P*=0.013, respectively). (E) Olfactory memory of electric shock learning was assessed after metamorphosis. Third-instar larvae underwent a five-cycle spaced training regimen (5× spaced training), with memory assessed in adults 3-4 days after eclosion. The animals were either given water or sucrose prior to training. Olfactory memory through metamorphosis was not detectable after either 1× training without (*n*=9) or with (*n*=12) sucrose feeding, nor after 5× spaced training (*n*=14; one-sample *t*-test with Holm-Sidak adjusted *P*-values; *P*=0.739, *P*=0.637, *P*=0.739, respectively). Data represent PI values shown as scatter dot plots, with each bar representing the mean±s.e.m. and each dot representing individual data points. **P<0.01, *P<0.05; not significant (ns), *P*≥0.05. Additional statistical details are provided in Table S1. The dots within the plots represent biological replicates (i.e. each dot is a separate larval/adult cohort).

To test this, we trained wild-type *D. melanogaster* of the Canton-S strain following the protocol described by Pauls et al. (2010b), using amyl acetate (AM) and benzaldehyde (BA) as conditioned stimuli (CS). An electric shock served as the aversive unconditioned stimulus (US). As previously reported (Pauls et al., 2010b), a single training trial consisting of a single CS-US pairing (Fig. 1A) elicited a significant aversive memory score when tested immediately after training (Fig. 1B). Pre-feeding larvae with sucrose prior to training resulted in a comparable memory score (Fig. 1B). Consistent with earlier work (Eschment et al., 2020), protein synthesis-dependent LTM in larvae can be detected 5 h after five spaced training trials with sucrose feeding, originally demonstrated with high salt as the aversive US. Here, we confirm that a comparable 5-h LTM can also be induced when electric shocks are used as the aversive US (Fig. 1D). However, when equivalently trained larvae were allowed to undergo metamorphosis, the resulting adults (3-4 days post eclosion) did not retain this memory. No significant memory expression was observed in adult flies (Fig. 1E).

**Fig. 2.**
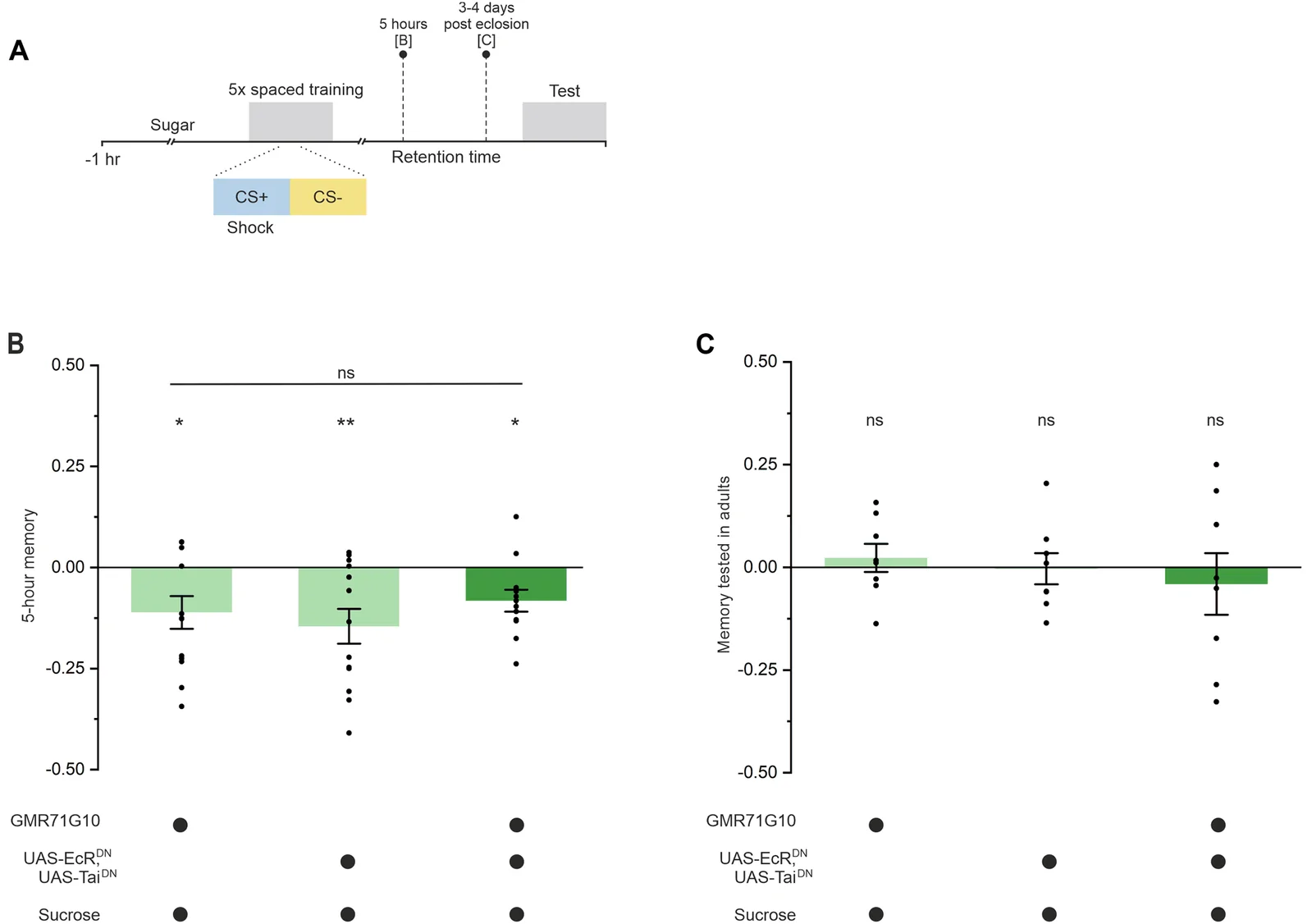
**No memory through metamorphosis in *D. melanogaster* with unpruned mushroom body γ lobes after olfactory electric shock conditioning.** (A) Schematic of five-cycle electric shock spaced training (5× spaced training) with 1-h sucrose feeding prior to training. The training consists of five cycles, each separated by a 15-min rest period. Memory was tested at different time points after training (retention time) as written in the scheme (with the relevant panels indicated). (B) *D. melanogaster* larvae expressing dominant-negative variants of both EcR and Tai driven by GMR71G10-Gal4 (*UAS-EcR^DN^/+; UAS-Tai^DN^/GMR71G10-Gal4*, *n*=12), as well as both control groups (*GMR71G10*-Gal4/+, *n*=13; or *UAS-EcR^DN^/+;UAS-Tai^DN^/+*, *n*=13) showed 5-h memory after 5× spaced electric shock training (one-sample *t*-test with Holm-Sidak adjusted *P*-values; *P*=0.023, *P*=0.017, *P*=0.023, respectively), which are not significantly different from each other (one-way ANOVA, *F*=0.681, *P*=0.513). (C) *D. melanogaster* larvae expressing dominant-negative variants of both EcR and Tai driven by GMR71G10-Gal4 do not retain memory through metamorphosis after 5× spaced electric shock training, despite lack of axon pruning, nor do control flies. Memory retention was not observed in adult flies of the unpruned γ lobe group (*UAS-EcR^DN^/+; UAS-Tai^DN^/GMR71G10-Gal4*, *n*=8) or in the control groups (*GMR71G10*-Gal4/+, *n*=8; or *UAS-EcR^DN^/+;UAS-Tai^DN^/+*, *n*=8; one-sample *t*-test with Holm-Sidak adjusted *P*-values; *P*=0.894, *P*=0.932, *P*=0.894, respectively). Data represent PI values shown as scatter dot plots, with each bar representing the mean±s.e.m. and each dot representing individual data points. **P<0.01, *P<0.05; ns, *P*≥0.05. Additional statistical details are provided in Table S1. The dots within the plots represent biological replicates (i.e. each dot is a separate larval/adult cohort).

Associative olfactory memories are stored at KC-to-MBON synapses in both larvae and adult flies (Thum and Gerber, 2019). During metamorphosis, the larval mushroom body undergoes stereotypic remodeling. We therefore asked whether memory maintenance might be possible if this remodeling process – characterized by axonal and dendritic pruning followed by regrowth – was genetically inhibited. To test this, we overexpressed in γ-KCs a dominant-negative mutant variant of the ecdysone receptor (EcR^DN^; Lee et al., 2000) – a key regulator of MB remodeling – together with a dominant-negative variant of its co-receptor Taiman (Tai^DN^; Bai et al., 2000), under control of the γ-KC-specific driver line GMR71G10-Gal4. This genetic manipulation has been shown to result in a severe pruning deficit of mushroom body γ-KCs, essentially preserving the larval γ-axonal lobes in the adult brain (Poppinga et al., 2022). Following olfactory conditioning with electric shock as an aversive US, larvae of this genotype, as well as genetic control strains (heterozygous Gal4 or UAS lines), showed significant 5-h memory following five spaced training trials with sucrose feeding (Fig. 2A,B). However, no memory retention was observed in adult flies (3-4 days after eclosion) that had been equivalently trained during their larval stage (Fig. 2C).

**Fig. 3.**
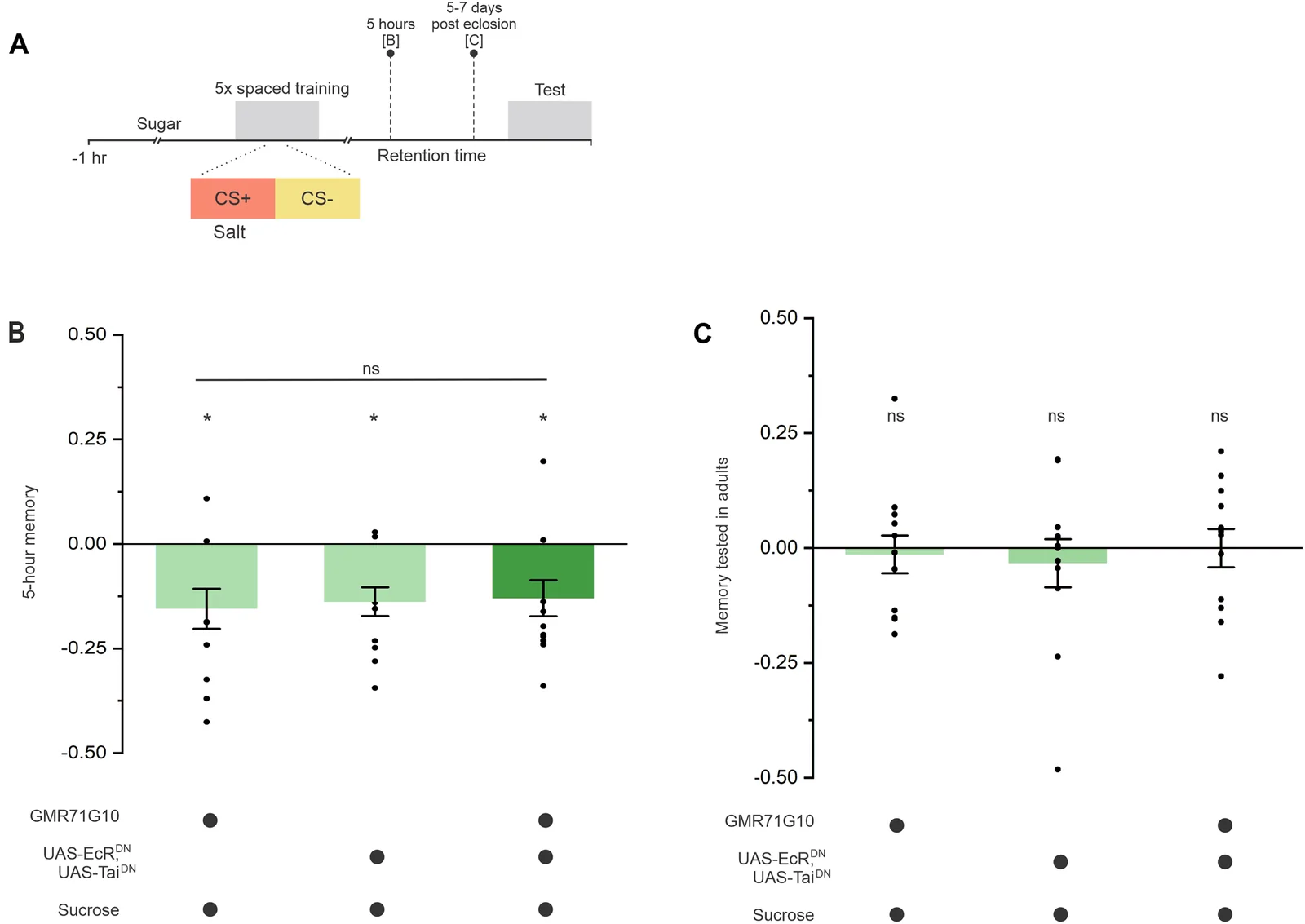
**No memory through metamorphosis in *D. melanogaster* with unpruned mushroom body γ lobes after odor-high salt conditioning.** (A) Schematic of five-cycle odor-high-salt training (5× spaced training), in which one odor was paired with high-salt (CS+) and the other was not (CS−). The training consists of five cycles, each separated by a 15-min rest period. Memory was tested at different time points after training (retention time) as written in the scheme (with the relevant panels indicated). (B) *D. melanogaster* larvae expressing dominant-negative variants of both EcR and Tai driven by GMR71G10-Gal4 (*UAS-EcR^DN^/+; UAS-Tai^DN^/GMR71G10-Gal4*, *n*=12) as well as both control groups (*GMR71G10*-Gal4/+, *n*=12; or *UAS-EcR^DN^/+;UAS-Tai^DN^/+*, *n*=12) showed 5-h memory after 5× spaced odor-high-salt training (one-sample *t*-test with Holm-Sidak adjusted *P*-values; *P*=0.015, *P*=6.169×10^−3^, *P*=0.015, respectively), which are not significantly different from each other (one-way ANOVA, *F*=0.092, *P*=0.91). (C) *D. melanogaster* larvae expressing dominant-negative variants of both EcR and Tai driven by GMR71G10-Gal4 do not retain memory through metamorphosis after 5× spaced odor-high-salt training, even though their γ-lobes were unpruned. Memory was not observed in the unpruned γ lobe group or in the control groups (one-sample *t*-test with Holm-Sidak adjusted *P*-values; *P*=0.935, *P*=0.903, *P*=0.993, respectively). A sample of the experimental group was dissected to validate and confirm lack of pruning. Data represent PI values shown as scatter dot plots, with each bar representing the mean±s.e.m. and each dot representing individual data points. *P<0.05; ns, *P*≥0.05. Additional statistical details are provided in Table S1. The dots within the plots represent biological replicates (i.e. each dot is a separate larval/adult cohort). See Fig. S1 for correlation of larval-adult dots (i.e. PI values).

We decided to repeat the experiment in another memory paradigm, which might include a more physiologically relevant aversive US. In fact, LTM has previously been demonstrated in larvae, using alternative CS odors (octanol and amyl acetate) and high salt as the aversive US (Eschment et al., 2020). Therefore, we repeated the experiments under equivalent conditions (Fig. 3A). Notably, in this set of experiments, larvae underwent olfactory conditioning together and later separated, with half tested for memory retention 5 h later, and the other half left to pupariate and tested as adult flies. Once again, control or pruning-deficient larvae exhibited significant 5-h LTM (Fig. 3B), yet no memory expression was detectable in adults (5-7 days after eclosion) that had been trained as larvae (Fig. 3C). Importantly, no significant correlation was observed between the memory performance of larvae and adult fly cohorts that underwent their training as larvae together (Fig. S1), further validating the lack of memory retention through metamorphosis.

Taken together, these findings indicate that aversive associative olfactory memories acquired during the larval stage are not maintained across metamorphosis in *D. melanogaster*, even under conditions in which mushroom body γ-KC remodeling is genetically suppressed.

## DISCUSSION

Pre-imaginal olfactory conditioning and the transfer of learning were first described in *D. melanogaster* by Thorpe (1939). In that study, larvae were raised on food containing peppermint oil, and the resulting adults showed a preference, rather than an aversion, to peppermint. Although the larvae and pupae were washed with distilled water, it could not be ruled out that residual traces of the scent remained on or within the animals during metamorphosis, potentially influencing the adult olfactory system, as proposed by the ‘chemical legacy hypothesis’ (Corbet, 1985). Non-associative effects due to mere odor exposure could also not be excluded. While some studies confirmed these findings (Hershberger and Smith, 1967), others failed to replicate them (Barron and Corbet, 1999).

Over the decades, research on learning and memory in insects has generally been approached from three perspectives. First, neuroethologists have asked in which ecological contexts learning confers an adaptive advantage. This led to the concept of ‘Hopkins’ host-selection principle’ (Hopkins, 1916), proposing that animals that successfully develop in a particular larval environment may later return to this context as adults to reproduce. However, this principle does not directly address whether adult behaviors are shaped by learning (Jaenike, 1983; Barron, 2001). Second, learning psychologists have investigated the principles governing learning processes through carefully controlled experiments. For example, Tully et al. (1994) demonstrated associative learning in larvae using aversive Pavlovian conditioning. Odor-shock associations during the larval stage elicited odor-specific memory retention in adults, whereas pure odor exposure or electric shock alone did not. Third, neurophysiologists have focused on the neural circuits and synaptic mechanisms underlying learning. In *D. melanogaster*, the CS pathway, US pathway, and their convergence are well described for both larvae and adults. Larvae perceive odors via the dorsal organ, while adults use the third antennal segment and maxillary palps (Gerber and Stocker, 2007). Although olfactory sensory neurons (OSNs) express different receptor subsets at each stage, some overlap exists, so differences in receptor expression alone do not preclude memory transfer (Kreher et al., 2005, 2008). However, OSNs degenerate during pupation, and second-order projection neurons also undergo pruning and regrowth, dissolving their larval synaptic connections. In the mushroom body, aversive US signals are mediated by subsets of DANs that innervate KC axons at stereotypic locations (Weiglein et al., 2021; Schleyer et al., 2020; Eschbach et al., 2020; Weber et al., 2025). It is now known that many DANs undergo extensive remodeling processes during metamorphosis, including pruning and regrowth – in some cases to targets outside of the mushroom body (a process known as trans-differentiation), while larval DANs of the primary protocerebral anterior medial (pPAM) cluster undergo programmed cell death (Truman et al., 2023). Although the KCs, which serve as the convergence point for CS and US pathways, encode memory at their synapses with MBONs in both larvae (Eichler et al., 2017; Eschbach et al., 2020) and adults (Aso et al., 2014), these intrinsic neurons themselves undergo axonal and dendritic pruning and regrowth during metamorphosis (Yaniv and Schuldiner, 2016). In fact, the entire mushroom body circuit undergoes large-scale stereotypic remodeling that does not maintain a single input-output synapse throughout metamorphosis and is thus expected to erase larval memory traces (Truman et al., 2023). Remarkably, even when pruning of the γ-KCs is genetically prevented, we observed no maintenance of larval memory into adulthood. The implications of inhibiting pruning of γ-KCs on the overall mushroom body circuitry remain poorly defined, and are the focus of an ongoing project in the Schuldiner laboratory. We have previously shown that cell-autonomous inhibition of γ-KC axon pruning prevents the pruning of the GABAergic anterior paired lateral (APL) neuron (Mayseless et al., 2018). However, we do not know if remodeling of other members of the mushroom body circuit, including DANs and MBONs, is similarly affected. Since prevalent models suggest that short-term memory, and likely also long-term memory (Séjourné et al., 2011), are encoded at the KC-MBON synapses, the loss of memory suggests that despite the genetic inhibition of γ-KC remodeling, larval KC-MBON synapses are not maintained into adulthood. Whether this is because of small-scale synapse elimination, normal MBON remodeling, or circuit connectivity that does not support memory retention/retrieval, remains to be determined.

We also considered whether differences in experimental setup could account for discrepancies with earlier reports. Tully et al. (1994) tested adults 2-3 days after eclosing, while in our experiments the flies were slightly older (3-4 or 5-7 days post eclosion). We therefore cannot rule out transient persistence of memory in adult flies shortly after eclosion, which was lost by the time of testing. Additionally, Tully et al. (1994) presented odors via laminar airflow and used eight training trials, whereas five spaced trials are now known to suffice for LTM formation in adult flies (Heisenberg et al., 1985) as well as in larvae (Eschment et al., 2020). Given that our protocols achieve comparable larval memory scores, it seems unlikely that these differences account for the lack of memory retention. In addition, we have expanded the learning regime by using high-molar salt as negative reinforce, which represents a more commonly used and potentially more physiologically and ecologically relevant cue (Niewalda et al., 2008).

It remains possible that memory retention across metamorphosis may occur for other forms of associative learning or in other insect species. For example, in Tenebrio beetles, the larval central complex is already established, and KC proliferation continues into adulthood, which may allow larval memories to persist. It might also be interesting to investigate whether maintenance of learned information throughout metamorphosis is more prevalent in hemimetabolous insects than in holometabolous ones. In *D. melanogaster*, however, we conclude that aversive associative olfactory memories formed during the larval stage are not maintained through metamorphosis. Notably, our experimental setup cannot distinguish between complete loss of consolidated memories during metamorphosis versus failure to retrieve these memories as adults. Importantly, our conclusion does not preclude the retention of other memory types or the existence of memory transfer in species with different developmental or neural architectures.

## MATERIALS AND METHODS

### Fly stocks

Fly strains were reared on standard *Drosophila* medium at 25°C, 60% humidity, with a 12-h light-dark cycle. The Canton-S strain was used as a wild-type control. To prevent pruning of the mushroom body γ-lobe, the effector line *UAS-EcR^DN^;UAS-Tai^DN^* (Cherbas et al., 2003; Jang et al., 2009; Poppinga et al., 2022) was specifically expressed in γ-KCs using the γ-specific driver line *GMR71G10*-Gal4 (Pfeiffer et al., 2008; Alyagor et al., 2018, obtained from Bloomington *Drosophila* Stock Center, #39604). Genetic controls were established by crossing the Gal4-driver line (*GMR71G10*-Gal4) or the UAS-effector line (*UAS-EcR^DN^;UAS-Tai^DN^*) to the *w^1118^* strain.

### Aversive olfactory conditioning in *D. melanogaster* larvae using electric shocks

Associative electric shock conditioning experiments in *D. melanogaster* larvae were performed as previously described in Pauls et al. (2010b) with some modifications. Briefly, experiments were conducted on assay plates (92-mm diameter, Sarstedt, Nümbrecht, 82.1472) filled with a thin layer of 2.5% w/v agarose (Sigma-Aldrich, 9012-36-6) containing 0.01 M lithium chloride (Merck Millipore, 105679) with attached copper electrodes. As olfactory stimuli, 10 μl of the odors amyl acetate [AM; Sigma-Aldrich, 46022, diluted 1:250 in paraffin oil (Sigma-Aldrich, 76235)] and benzaldehyde (BA; undiluted, Sigma-Aldrich, 418099) were loaded into custom-made Teflon containers (4.5-mm diameter) with perforated lids. To train larvae, groups of approximately 30 third-instar larvae (L3), 5-6 days after egg laying (with a range variability of 24 h), were exposed for 1 min to each odor with a break of 90 s between the two odor applications. One odor was temporally paired during the last 30 s of odor onset with an electric shock (90 V, direct current) (CS+), whereas the other odor was presented without the shock (CS−). Larvae underwent either a single cycle of electric shock conditioning (1× training) or a five-cycle training regimen, with 15-min rest intervals on agarose plates between cycles (5× spaced training).

### Assessment of olfactory learning and memory after electric shock conditioning in *D. melanogaster* larvae

Learning and memory after electric shock conditioning were assessed in *D. melanogaster* larvae by transferring the trained larvae (approximately 30 per cohort), following a defined retention period – either immediate (Fig. 1B) or 5 h after training (Fig. 1D and Fig. 2B) – onto fresh agarose plates containing 0.01 M lithium chloride mixed in 2.5% agarose. On these plates, the odors (AM diluted 1:250 and undiluted BA, as in the training) were presented on opposite sides. After 5 min, the number of individuals located on the AM side, BA side, or within a 1-cm central neutral zone was recorded. The preference index was calculated by subtracting the number of larvae on the side associated with the CS− from the number on the side with the CS+ and dividing this by the total number of larvae counted. To measure the effect of associative learning, a performance index (PI) was then calculated by averaging the preference indices from two reciprocal experiments in which the roles of the CS+ and CS− were reversed.

### Assessment of olfactory memory through metamorphosis after electric shock conditioning in *D. melanogaster* adult flies

To assess memory retention through metamorphosis, larvae were transferred to standard food vials immediately after training and kept in a 25°C incubator with a 12/12 h light-dark cycle. Notably, these larvae (approximately 30 per group), which were tested as adults, underwent electric shock conditioning separately from the larvae that were tested 5 h after training (as detailed in the previous section). After eclosion, adult flies (3-4 days old) were transferred into the T-maze part of a custom-built T-maze apparatus (Schwaerzel et al., 2002) and allowed to habituate for 60 s. The flies were then simultaneously presented with CS+ and CS− odors from different arms of the maze for 2 min with a constant airflow (±167 ml/min). AM was diluted 1:250, as in the larval testing. BA (Sigma-Aldrich, M8410) was diluted 1:100 in mineral oil, in contrast to the larval training and testing (in which it was undiluted), since unlike in larvae, in adult flies it is a strong repellent. The number of flies in each arm was counted. A preference index was calculated by subtracting the number of flies in the CS− arm from the number in the CS+ arm and dividing by the total number of flies tested. PI values were determined by averaging the preference indices from two reciprocal training sessions in which the odors used as CS+ and CS− were exchanged.

### Odor-high salt conditioning in *D. melanogaster* larvae

Associative odor-high-salt conditioning experiments in *D. melanogaster* larvae were performed as previously described in Widmann et al. (2016) with some modifications. Briefly, experiments were conducted on assay plates (85-mm diameter) filled with a thin layer of 1% agarose containing either pure agarose or agarose plus 3 M sodium chloride. AM diluted 1:250 in paraffin oil and undiluted 1-octanol (Sigma-Aldrich, 111-87-5) were used as olfactory stimuli, each dispensed in a volume of 10 μl into custom-made Teflon containers (4.5-mm diameter) equipped with perforated lids. Groups of approximately 60 L3 larvae, 5-6 days after egg laying (with a range variability of 24 h), were exposed to each odor for 5 min; one odor was paired with sodium chloride (CS+) and the other was presented without (CS−). Larvae underwent a five-cycle training regimen, with 15-min rest intervals on agarose plates between cycles (5× spaced training). Notably, unlike with the electric shock conditioning, here the tested larvae and adults were trained together – i.e. out of the 60 larvae per group that underwent high-salt conditioning, half were tested for memory as larvae 5 h later, while the other half were left to pupariate and eclose before being tested as adults (see details below).

### Assessment of olfactory learning and memory after high salt conditioning in *D. melanogaster* larvae

Learning and memory after odor-high-salt conditioning were assessed in *D. melanogaster* larvae by transferring them, 5 h after their training, onto fresh agarose plates containing 3 M sodium chloride mixed in 1% agarose. On these plates, the odors (AM 1:250 and undiluted 1-octanol, as in the training) were presented on opposite sides. After 5 min, the number of individuals located on both odor sides, or within a 1-cm central neutral zone was recorded. The preference index was calculated by subtracting the number of larvae on the side associated with the CS− from the number on the side with the CS+ and dividing this by the total number of larvae counted. To measure the effect of associative learning, a PI was then calculated by averaging the preference indices from two reciprocal experiments in which the roles of the CS+ and CS− were reversed.

### Assessment of olfactory memory through metamorphosis after high salt conditioning in *D. melanogaster* adult flies

To assess memory retention through metamorphosis, larvae were transferred to standard food vials immediately after training and kept in a 25°C incubator with a 12/12 h light-dark cycle. After eclosion, adult flies (5-7 days old) were transferred into the T-maze part of a designed T maze (MazeEngineers™) and allowed to habituate for 60 s. The flies were then simultaneously presented with CS+ and CS− odors with the same concentrations used for their larval training (1:250 AM and undiluted 1-octanol, as in the larval training and testing), from different arms of the maze for 90 s with a constant airflow (±170 ml/min). The number of flies in each arm was counted. A preference index was calculated by subtracting the number of flies in the CS− arm from the number in the CS+ arm and dividing by the total number of flies tested. PI values were determined by averaging the preference indices from two reciprocal training sessions in which the odors used as CS+ and CS− were exchanged.

## Supplementary Material



10.1242/biolopen.062512_sup1 Supplementary information
