# Supplementary material for: No evidence for aversive associative olfactory memory through metamorphosis in Drosophila
Source: Biol Open. 2026 Aug 7;15(8):bio062512. doi: 10.1242/bio.062512 (PMC13488412; doi:10.1242/bio.062512)
Supplement: Supplementary file 1 [file biolopen-15-062512-s1.pdf]

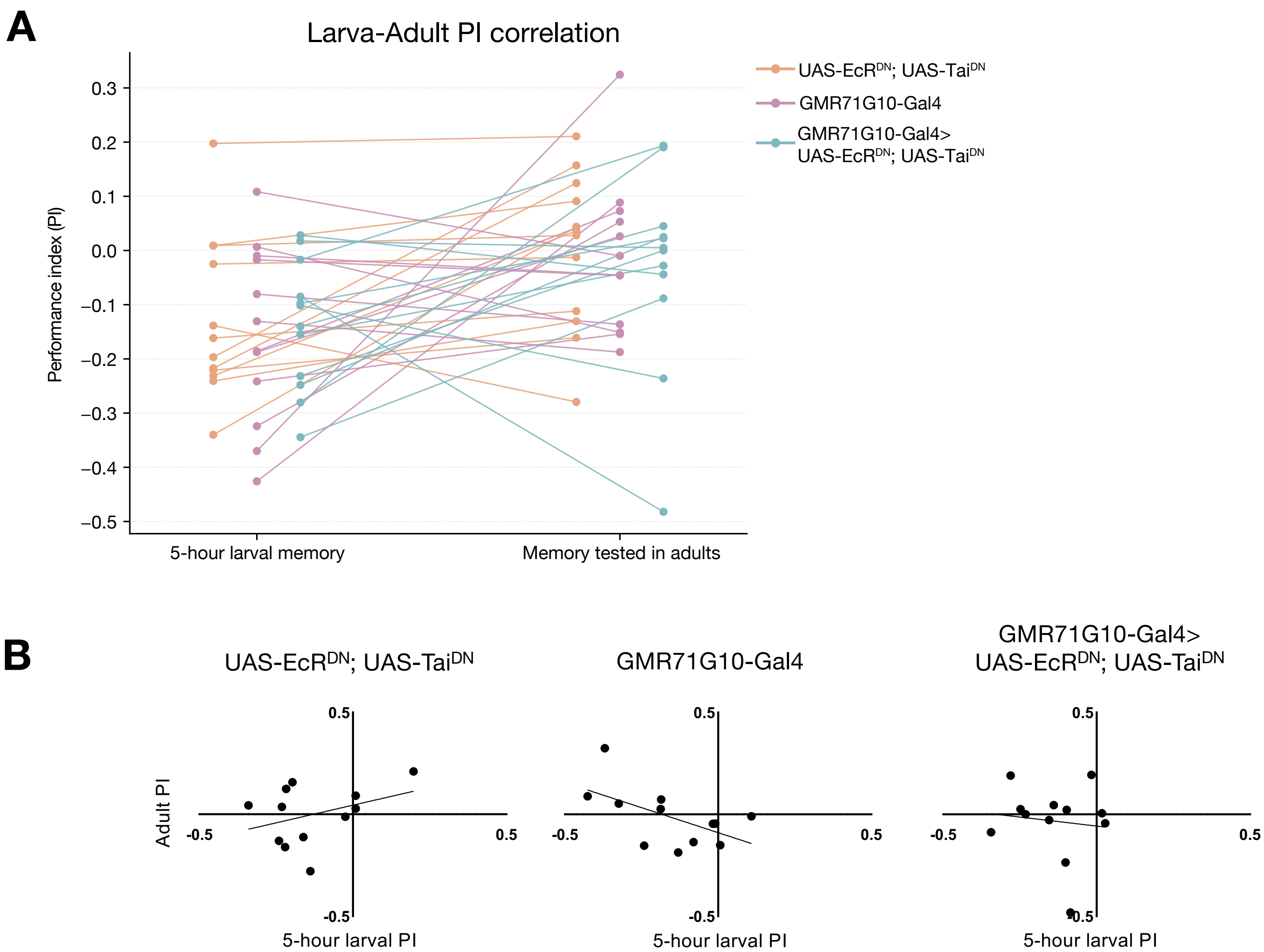

Pearson r	UAS-EcR^{DN}; UAS-Tai^{DN}	GMR71G10-Gal4	GMR71G10-Gal4> UAS-EcR^{DN}; UAS-Tai^{DN}
r	0.3573	-0.5680	-0.1158
95% confidence interval	-0.2724 to 0.7728	-0.8612 to 0.008775	-0.6467 to 0.4907
R squared	0.1277	0.3226	0.01340
**P (two-tailed)**	**0.2541**	**0.0540**	**0.7201**
n of XY Pairs	12	12	12

Fig. S1. No correlation of larval-to-adult performance - related to Figure 3.

A: Spaghetti plot describing the correlation between the PI values of larvae 5 hours after training, and adults 5-7 days after eclosion, which were trained together as larvae, with the experimental and control groups marked in different colors.

B: Scatter plots illustrating Perason’s correlation coefficient (r) between the PI of larvae 5 hours after training and adults 5-7 days after eclosion, which were trained together as larvae, in each of the 3 genotypes. Statistical analysis is presented in the table below.

Biology Open • Supplementary information

Table S1. Sample size (n), mean±s.e.m. and statistical details of test against chance level.

	Group[1]	n	Mean±s.e.m	Normal distri-bution[2]	Shapiro-Wilk test	One-sample t-test [3]	p value[4]	Signi-ficant[5]
Fig 1B	1	10	0.201±0.049	yes	W=0.901, p=0.227	t=4.052, df=9	0.0058	*
	2	10	-0.183±0.052	yes	W=0.981, p=0.969	t=3.508, df=9	0.0066	*
Fig 1D	1	10	-0.041±0.039	yes	W=0.874, p=0.112	t=1.038, df=9	0.5465	ns
	2	12	-0.017±0.055	yes	W=0.946, p=0.579	t=0.3157, df=11	0.7582	ns
	3	16	-0-101±0.030	yes	W=0.967, p=0.797	t=3.337, df=15	0.0134	*
Fig 1E	1	9	-0.033±0.061	yes	W=0.942, p=0.598	t=0.5374, df=8	0.7390	ns
	2	12	-0.053±0.046	yes	W=0.902, p=0.167	t=1.165, df=11	0.6089	ns
	3	14	-0.030±0.042	yes	W=0.929, p=0.296	t=0.7118, df=13	0.7390	ns
Fig 2B	1	13	-0.111±0.041	yes	W=0.896, p=0.117	t=2.732, df=12	0.0231	ns
	2	13	-0.145±0.043	yes	W=0.904, p=0.150	t=3.363, df=12	0.0167	ns
	3	12	-0.082±0.094	yes	W=0.948, p=0.605	t=3.021, df=11	0.0231	ns
Fig 2C	1	8	0.023±0.034	yes	W=0.971, p=0.906	t=0.6651, df=7	0.8944	ns
	2	8	-0.003±0.038	yes	W=0.939, p=0.604	t=0.08916, df=7	0.9315	ns
	3	8	-0.041±0.075	yes	W=0.949, p=0.705	t=0.5394, df=7	0.8944	ns
Fig 3B	1	12	-0.155±0.048	yes	W=0.960, p=0.900	t=3.249, df=11	0.0154	*
	2	12	-0.138±0.034	yes	W=0.958, p=0.761	t=4.007, df=11	0.0062	*
	3	12	-0.130±0.043	yes	W=0.914, p=0.240	t=3.011, df=11	0.0154	*
Fig 3C	1	12	-0.014±0.041	yes	W=0.908, p=0.204	t=0.3344, df=11	0.9346	ns
	2	12	-0.033±0.052	yes	W=0.861, p=0.05	t=0.6315, df=11	0.9030	ns
	3	12	-0.000±0.042	yes	W=0.965, p=0.845	t=0.0085, df=11	0.9933	ns

[1]Numbers correspond to boxplots from left to right. [2]Significance level was set to α=0.05, p≥0.05 indicates normal distribution. [3]One-sample t-test; Theoretical mean was 0.000. [4]Holm-Šidák-corrected p value, significance level equates to α=0.05. [5]ns indicates p≥0.05, * indicates p<0.05.

Biology Open • Supplementary information
