# Supplementary material for: No evidence for aversive associative olfactory memory through metamorphosis in Drosophila
Source: Biol Open. 2026 Aug 7;15(8):bio062512. doi: 10.1242/bio.062512 (PMC13488412; doi:10.1242/bio.062512)
Supplement: Supplementary file 2 [file biolopen-15-062512_review_history.pdf]

No evidence for aversive associative olfactory memory through metamorphosis in Drosophila

Haiko Poppinga, Rotem Mika, Maria Schwartz, Oren Schuldiner, André Fiala and Hagar Meltzer

10.1242/bio.062512

Editor: Sandhya Koushika

Review timeline

Original submission: 4 February 2026
Editorial decision: 11 February 2026
First revision received: 8 July 2026
Accepted: 14 July 2026

Original submission

First decision letter

MS ID#: bio.062512

MS Title: No evidence for aversive associative olfactory memory through metamorphosis in Drosophila

Authors: Haiko Poppinga, Rotem Mika, Maria Schwartz, Oren Schuldiner, André Fiala and Hagar Meltzer

I have now reached a decision on the above manuscript.

The reviewer reports are shown at the bottom of this email.

As you will see, the reviewers gave favourable reports, but raised some critical points that will require amendments to your manuscript. I hope that you will be able to carry these out, because we would like to be able to accept your paper.

At this stage, we also ask you to ensure your manuscript complies with our formatting guidelines – please see our manuscript preparation guidelines for details. Provided you are able to fully address the referees' comments, we are positive about publication of your paper (we accept over 95% of revision submissions) and therefore hope you won't mind any extra work involved in reformatting your manuscript at this point.

Please upload both a 'clean' version of your Word file, along with a highlighted version clearly showing where you have made changes in the revised manuscript. Please avoid using 'Track changes' in Word files as these are lost in PDF conversion.

I should be grateful if you would also provide a point-by-point response detailing how you have dealt with the points raised by the reviewers in the 'Response to Reviewers' box. Please attend to all of the reviewers' comments. If you do not agree with any of their criticisms or suggestions please explain clearly why this is so.

Reviewer 1

© 2026. Published by The Company of Biologists under the terms of the Creative Commons Attribution License (https://creativecommons.org/licenses/by/4.0/).

Comments for the author

In this study, the authors revisit the long-standing question of whether larval memories persist through metamorphosis in holometabolous insects. By leveraging a standard, well-characterized classical aversive olfactory conditioning assay and established underlying circuit mechanisms in Drosophila, the study shows that larval memories do not survive into adulthood, contrary to several previous reports. The simple experimental design provides behavioral evidence that larval aversive olfactory memories are erased during metamorphosis, even when remodeling of the mushroom body-a critical learning center-is largely prevented. Overall, this study reopens a long-standing controversy in the field and paves the way for examining whether similar principles apply to other forms of memory and across species.

The manuscript is concise, clearly written, and well organized. Although the data support the authors' conclusion, several concerns regarding the interpretation require further clarifications. For instance:
1. Please specify the retention time explicitly in both schematic and the Methods section.
2. It is unclear whether the same cohort of larvae (n=30) used for training was transferred, cultured, and subsequently tested for adult performance, or whether different cohorts were pooled. This required clarification.
3. Given the variability in learning and memory performance within groups/genotypes, it would be informative to examine a one-to-one correlation between larval and adult performance. Pooling data may obscure subtle effects-did larval batches that performed strongly also perform better as adults?
4. The Methods state: "To train larvae, groups of 30 larvae (6 days old) were exposed……" were larvae synchronized for age? Explicitly indicate the larval stage used for training. Can the variability in developmental stage contribute to the observed variability in performance?
5. While the data suggest that larval memories do not persist, the experiments cann't distinguish between complete erasure of consolidated memory and/or failure of retrieval in adults. This distinction should be discussed more carefully. Additionally, was the conditioning paradigm sufficient to induce long-lasting memory in larvae that persist metamorphosis?
6. Adult testing was performed at 5-7 days post-eclosion. What was the rationale for selecting this time window? Were earlier time points examine to rule out transient persistence of memory shortly after eclosion?
7. Although genetic tools were used to suppress the pruning and regrowth of mushroom body Kenyon cells, it remains unclear whether the circuit- or synaptic-level remodeling in downstream neurons persists during metamorphosis and could account for memory loss.
8. The manuscript does not clearly specify the odor concentrations used for assessing olfactory learning and memory in larvae and adults. Were the same concentrations used at both stages? If different concentrations were applied, could this influence the behavioral outcome or comparability between larval and adult performance? Clarification is needed, as odor intensity can significantly affect conditioning strength and behavioral readouts.

Minor comments:
1. The authors may consider using keywords: Neuronal remodeling, larval memory, and olfactory conditioning.
2. After first usage, Drosophila melanogaster should be abbreviated as D. melanogaster.
3. Please follow standard genotype formatting consistently throughout the manuscript.
4. The usage of hypen (-), en dash (-), and em dash (—-) should be standardized and applied appropriately.
5. Lines 33-40 on page 8 are unclear and would benefit from rephrasing.
6. The terms "Kenyon cells" and "KC" are used interchangeably.
7. The manuscript should be carefully checked for typographical errors.

<u>Reviewer 2</u>

Comments for the author

This manuscript revisits a long-standing question with a controversial history in the literature: whether larval olfactory memories persist through metamorphosis in Drosophila melanogaster. Using two aversive conditioning paradigms (electric shock and high salt), as well as both wild-type

© 2026. Published by The Company of Biologists under the terms of the Creative Commons Attribution License (https://creativecommons.org/licenses/by/4.0/).

and pruning-defective γ-Kenyon cell flies, the authors find no evidence for memory retention into adulthood. The study is carefully designed and directly addresses the work of Tully et al. (1994) using modern genetic and experimental tools.

1. Experimental Quality

Appropriate genetic and training controls were performed. Two independent aversive olfactory paradigms were used. The statistical analyses appear appropriate.
Major concern:
a) Adult flies were not tested for their ability to acquire memory of these cues as adults. Including such a control would strengthen the interpretation that the assay is sufficiently sensitive to detect memory under the testing conditions.
b) Please report n directly in each figure legend to improve clarity and transparency (currently reported only in a supplemental table).

2. Reproducibility
The methods are well described.
Major concern:
c) It is unclear whether each dot in the scatter plots represents an independent biological replicate (e.g., separate larval cohorts) or technical replicates of the same cohort. This should be explicitly clarified.
3. Completeness
Major concern:
The main conclusion is that "associative olfactory memories are not maintained through metamorphosis." However, the authors test only aversive olfactory long-term memory and do not examine appetitive (positive) olfactory memory. While they state that their conclusion "does not preclude the retention of other memory types," the title and framing suggest a broader generalization than is experimentally supported.
Given that other studies have reported evidence to the contrary, I suggest modifying the title to more precisely reflect the scope of the data. For example:
"Associative aversive olfactory memory is not maintained through metamorphosis in Drosophila."
4. Scholarship
The manuscript is well written and provides a thorough and thoughtful engagement with the relevant literature.

Reviewer's Responses to Questions

Experimental quality
Does each figure have the proper controls?
If 'No', please indicate reasons in Comments for Author box below.
Reviewer #1:
- Yes

Reviewer #2:
- No

Were the data analyzed using appropriate statistical tests?
If 'No', please indicate reasons in Comments for Author box below.
Reviewer #1:
- Yes

Reviewer #2:
- Yes

Reproducibility
Were experiments performed using adequate number of biological replicates?
If 'No', please indicate reasons in Comments for Author box below.

© 2026. Published by The Company of Biologists under the terms of the Creative Commons Attribution License (https://creativecommons.org/licenses/by/4.0/).

Reviewer #1:

- Yes

Reviewer #2:

- Yes

Does the methods section provide sufficient detail to permit reproducibility?
If 'No', please indicate reasons in Comments for Author box below.
Reviewer #1:

- No

Reviewer #2:

- Yes

Completeness
Are the manuscript's conclusions supported by the data?
If 'No', please indicate reasons in Comments for Author box below.
Reviewer #1:

- Yes

Reviewer #2:

- Yes

Scholarship
Do the authors cite and discuss the merits of data that would argue for and against their conclusion?
If 'No', please indicate reasons in Comments for Author box below.
Reviewer #1:

- Yes

Reviewer #2:

- Yes

Does the manuscript title & abstract accurately reflect the contents of the manuscript, without hyperbole?
If 'No', please indicate reasons in Comments for Author box below.
Reviewer #1:

- Yes

Reviewer #2:

- No

First revision

Author response to reviewers' comments

Comments from the Reviewers (response in blue)

Reviewer 1: In this study, the authors revisit the long-standing question of whether larval memories persist through metamorphosis in holometabolous insects. By leveraging a standard, well-characterized classical aversive olfactory conditioning assay and established underlying circuit mechanisms in Drosophila, the study shows that larval memories do not survive into adulthood, contrary to several previous reports. The simple experimental design provides

© 2026. Published by The Company of Biologists under the terms of the Creative Commons Attribution License (https://creativecommons.org/licenses/by/4.0/).

behavioral evidence that larval aversive olfactory memories are erased during metamorphosis, even when remodeling of the mushroom body-a critical learning center-is largely prevented. Overall, this study reopens a long-standing controversy in the field and paves the way for examining whether similar principles apply to other forms of memory and across species.

The manuscript is concise, clearly written, and well organized. Although the data support the authors' conclusion, several concerns regarding the interpretation require further clarifications.

We thank the reviewer for the positive assessment of our work and appreciate the constructive comments and suggestions. Our detailed responses are provided below.

1. Please specify the retention time explicitly in both schematic and the Methods section.

The exact retention times for the different experimental setups - immediate / 5-hours after training / 3-4 or 5-6 days after eclosion - are now clearly specified in the schemes in Figures 1-3 (along with the relevant panels indicated), as well as in the methods section.

2. It is unclear whether the same cohort of larvae (n=30) used for training was transferred, cultured, and subsequently tested for adult performance, or whether different cohorts were pooled. This required clarification.

We thank the reviewer for pointing out this issue, which we indeed did not properly explain. The reply differs between the two types of aversive stimuli used in the manuscript. For the experiment conducted with electric shock as the aversive stimulus, different groups of ~30 larvae were trained and then tested *either* 5 hours later (as larvae) *or* as adults (3-4 days after eclosion). In contrast, when salt was used as the aversive stimulus, groups of ~60 larvae were trained together, and then for each group, half of the larvae (~30) were tested 5 hours later as larvae, while the other half (~30) were left to pupariate and tested as adults 5-7 days after eclosion. This was now clarified in the text in both the results and methods sections (lines 141-143; 266-269; 290-293).

3. Given the variability in learning and memory performance within groups/genotypes, it would be informative to examine a one-to-one correlation between larval and adult performance. Pooling data may obscure subtle effects-did larval batches that performed strongly also perform better as adults?

We thank the reviewer for raising this important point. As explained in the previous comment, correlating larval-to-adult performance was only possible in the experiments in which high salt was used as the aversive stimuli, since larvae and adult flies underwent training together. We tested this and could not detect statistically significant larval-adult PI correlation in any of the groups - further strengthening the lack of memory retention through metamorphosis. This was now addressed in the results section (lines 146-148) as well as added as Supplemental Fig. 1, which contains a spaghetti plot highlighting larval-adult PI connections for all genotypes, as well as scatterplots illustrating Pearson's correlation coefficient for each genotype separately. Notably, the only group that displayed a p-value nearing significance (0.054 for the GMR71G10-Gal4 control group) showed inverse correlation, which is in fact an opposite trend of what we would expect had memory been retained through metamorphosis.

4. The Methods state: "To train larvae, groups of 30 larvae (6 days old) were exposed……" were larvae synchronized for age? Explicitly indicate the larval stage used for training. Can the variability in developmental stage contribute to the observed variability in performance?

The vials were flipped daily throughout the experiments, so that all larvae within a given cohort resulted from eggs laid on the same day, and picked as 3rd instar larvae (L3) after 5-6 days (i.e., the stage variability is within the range of 24 hours). This was now clarified in the methods section (lines 240-241 and 284-285).

5. While the data suggest that larval memories do not persist, the experiment can't distinguish between complete erasure of consolidated memory and/or failure of retrieval in adults. This distinction should be discussed more carefully. Additionally, was the conditioning paradigm sufficient to induce long-lasting memory in larvae that persist metamorphosis?

© 2026. Published by The Company of Biologists under the terms of the Creative Commons Attribution License (https://creativecommons.org/licenses/by/4.0/).

The reviewer is absolutely right that with our experimental setup we cannot distinguish between these two options, and we have now addressed this in the discussion (lines 221- 222). Regarding the second point, it was previously shown that 5 spaced trials are sufficient to induce long-term memory in larvae (Eschment et al., 2020, as stated in lines 118-120 and 209, and also repeated in the present study). However, we cannot rule out that the lower number of trials underlies the discrepancies with earlier reports (since 8 training trials were used in Tully et al. 1994), as clarified in the discussion.

6. Adult testing was performed at 5-7 days post-eclosion. What was the rationale for selecting this time window? Were earlier time points examine to rule out transient persistence of memory shortly after eclosion?

As we now clarified in the methods section, in our high salt experiments, we tested flies 5-7 days post eclosion (mainly for technical/convenience considerations), while for the electric shock experiments flies were a bit younger - 3-4 days post eclosion. However, in both cases, flies were still a bit older than those tested in Tully et al. (which were 2-3 days post eclosion), and therefore we indeed cannot rule out transient persistence of memory shortly after eclosion. This point was now clarified in the discussion (lines 204-206).

7. Although genetic tools were used to suppress the pruning and regrowth of mushroom body Kenyon cells, it remains unclear whether the circuit- or synaptic-level remodeling in downstream neurons persists during metamorphosis and could account for memory loss.

The reviewer is absolutely right and we now further elaborated in the discussion that with the exception of the APL neuron (Mayseless et al., 2018), the overall circuitry implications of inhibiting γ-KC pruning - and specifically the effects on DAN/MBON remodeling - are unknown, and are actually the focus of an ongoing project in the Schuldiner lab. We further emphasized that since prevalent models suggest that memory is encoded at the KC-MBON synapses, its loss suggests that larval KC-MBON synapses are not maintained into adulthood, despite the genetic inhibition of γ-KC remodeling. It remains to be determined whether this is because of small-scale synapse elimination, normal MBON remodeling, or circuit connectivity that does not support memory retention/retrieval (lines 192-202).

8. The manuscript does not clearly specify the odor concentrations used for assessing olfactory learning and memory in larvae and adults. Were the same concentrations used at both stages? If different concentrations were applied, could this influence the behavioral outcome or comparability between larval and adult performance? Clarification is needed, as odor intensity can significantly affect conditioning strength and behavioral readouts.

For the high-salt experiments yes, the exact same concentrations were used for larval and adult testing (1-octanol was undiluted and amyl acetate was diluted 1:250 in paraffin oil). For the electric shock experiments, amyl acetate was used in the same concentration for larval and adult testing (1:250), however benzaldehyde (BA) was used in different concentrations - undiluted for larval training as well as testing, yet diluted 1:100 for adult testing. This is since while BA is attractive in larvae, in adult flies it is a very strong repellent. When we trained and tested adult Canton-S flies, no aversive learning was measurable when BA was undiluted, but it became significant when using a 1:100 dilution. This was now clarified in the text (without showing the data; lines 273-275), and presented below as Reviewer Figure 1. The odor concentrations are now fully detailed in the methods section (lines 240-242, 255-256, 272-273, 284-285, 298, 312-313).

Minor comments:

1. The authors may consider using keywords: Neuronal remodeling, larval memory, and olfactory conditioning.

We thank the reviewer for this suggestion, we now added these keywords along with several others.

2. After first usage, Drosophila melanogaster should be abbreviated as D. melanogaster.

© 2026. Published by The Company of Biologists under the terms of the Creative Commons Attribution License (https://creativecommons.org/licenses/by/4.0/).

Done, thanks.

3. Please follow standard genotype formatting consistently throughout the manuscript.

We are not absolutely sure what the reviewer was referring to in this comment, however we now added the full genotypes of the experimental and control flies in the legends of Figures 2 and 3 and better clarified them in the methods section.

4. The usage of hypen (-), en dash (-), and em dash (—-) should be standardized and applied appropriately.

Done, thanks.

5. Lines 33-40 on page 8 are unclear and would benefit from rephrasing.

Thank you, we rephrased this part, hopefully it is now clearer (lines 181-184).

6. The terms "Kenyon cells" and "KC" are used interchangeably.

Thanks, we now changed all to KC (after the first use).

7. The manuscript should be carefully checked for typographical errors.

Done, thanks.

Reviewer 2: This manuscript revisits a long-standing question with a controversial history in the literature: whether larval olfactory memories persist through metamorphosis in Drosophila melanogaster. Using two aversive conditioning paradigms (electric shock and high salt), as well as both wild-type and pruning-defective γ-Kenyon cell flies, the authors find no evidence for memory retention into adulthood. The study is carefully designed and directly addresses the work of Tully et al. (1994) using modern genetic and experimental tools.

We thank the reviewer for the favorable assessment of our study and appreciate the constructive comments - our detailed responses are provided below.

1. Experimental Quality

Appropriate genetic and training controls were performed. Two independent aversive olfactory paradigms were used. The statistical analyses appear appropriate.

Major concern:

a) Adult flies were not tested for their ability to acquire memory of these cues as adults. Including such a control would strengthen the interpretation that the assay is sufficiently sensitive to detect memory under the testing conditions.

The reviewer is right that this control was not included. However, in Poppinga et al., 2022 we reported that flies from the same experimental genotype used in the current study (*UAS-EcR^{DN}/+; UAS-Tai^{DN}/GMR71G10-Gal4*) are capable of forming (short-term) aversive associative learning, although slightly reduced compared to controls (*GMR71G10*-Gal4/+ or *UAS-EcR^{DN}/+;UAS-Tai^{DN}/+*). Furthermore, we also tested the experimental and control genotypes for the ability to form long-term memory using electric shock as the aversive stimuli. While this assay was performed with different odors than those used in this study – methylcyclohexanol and 3-octanol – it still demonstrates the ability of these flies to form long-term aversive memory, using the same experimental apparatus. We are adding these data as Reviewer Figure 2, and would be happy to also include it as a Supplemental Figure in the manuscript if the reviewer sees fits. Notably, we are currently unable to repeat this experiment with the same odors used in the study, since the students that led the project - Haiko and Mika - are no longer in the Fiala and Schuldiner labs,

© 2026. Published by The Company of Biologists under the terms of the Creative Commons Attribution License (https://creativecommons.org/licenses/by/4.0/).

respectively (and, in the case of the Schuldiner lab, the lab and equipment were completely demolished by an Iranian missile on June 2025).

b) Please report n directly in each figure legend to improve clarity and transparency (currently reported only in a supplemental table).

We thank the reviewer for pointing this out and now added the n to the figure legends.

2. Reproducibility

The methods are well described.

Major concern:

c) It is unclear whether each dot in the scatter plots represents an independent biological replicate (e.g., separate larval cohorts) or technical replicates of the same cohort. This should be explicitly clarified.

The dots within the plots represent biological replicates, i.e., separate larval/adult cohorts. This was now clarified in the figure legends.

3. Completeness

Major concern:

The main conclusion is that "associative olfactory memories are not maintained through metamorphosis." However, the authors test only aversive olfactory long-term memory and do not examine appetitive (positive) olfactory memory. While they state that their conclusion "does not preclude the retention of other memory types," the title and framing suggest a broader generalization than is experimentally supported.

Given that other studies have reported evidence to the contrary, I suggest modifying the title to more precisely reflect the scope of the data. For example:

"Associative aversive olfactory memory is not maintained through metamorphosis in Drosophila."

We agree with the reviewer and appreciate the suggestion - we now added the word ‘aversive’ to the title of the manuscript (as well as emphasized the fact that it’s aversive memory in the abstract, summary statement, and concluding paragraph of the results section).

4. Scholarship

The manuscript is well written and provides a thorough and thoughtful engagement with the relevant literature.

Two figures provided to the referees in confidence have been removed.

Second decision letter

MS ID#: bio.062512R1

MS Title: No evidence for aversive associative olfactory memory through metamorphosis in Drosophila

Authors: Haiko Poppinga, Rotem Mika, Maria Schwartz, Oren Schuldiner, André Fiala and Hagar Meltzer

© 2026. Published by The Company of Biologists under the terms of the Creative Commons Attribution License (https://creativecommons.org/licenses/by/4.0/).

I am happy to tell you that your manuscript has been accepted for publication in Biology Open, pending our standard publication integrity checks. It was accepted on 14th July 2026.

© 2026. Published by The Company of Biologists under the terms of the Creative Commons Attribution License (https://creativecommons.org/licenses/by/4.0/).
